# Homelessness during COVID-19: challenges, responses, and lessons learned from homeless service providers in Tippecanoe County, Indiana

**DOI:** 10.1186/s12889-021-11687-8

**Published:** 2021-09-10

**Authors:** Natalia M. Rodriguez, Alexa M. Lahey, Justin J. MacNeill, Rebecca G. Martinez, Nina E. Teo, Yumary Ruiz

**Affiliations:** 1 Department of Public Health, College of Health and Human Sciences, Purdue University, West Lafayette, Indiana USA; 2 Weldon School of Biomedical Engineering, College of Engineering, Purdue University, West Lafayette, Indiana USA; 3 Department of Pharmacy Practice, College of Pharmacy, Purdue University, West Lafayette, Indiana USA; 4 Department of Anthropology, College of Liberal Arts, Purdue University, West Lafayette, Indiana USA

**Keywords:** COVID-19, Homelessness, Health disparities, Community-based participatory research, Socio-ecological model, Disaster response, Pandemic response

## Abstract

**Background:**

The COVID-19 pandemic laid bare some of the United States’ most devastating health and social inequities faced by people experiencing homelessness. Homeless populations experience disproportionate rates of underlying health conditions, stigma and marginalization that often disenfranchise them from health and social services, and living conditions that potentiate the risk of COVID-19 transmission and adverse outcomes.

**Methods:**

Guided by the socio-ecological model, this community-based participatory research study examined the impacts of the COVID-19 public health crisis on people experiencing homelessness in Tippecanoe County, Indiana, and the ways in which homeless service providers prepared for, experienced, and responded to the pandemic. Eighteen (18) semi-structured interviews were conducted with representatives of 15 community-based organizations, including shelters and other homeless service providers.

**Results:**

Qualitative content analysis revealed myriad challenges at the individual and interpersonal levels faced by people experiencing homelessness as a result of the pandemic, and multilevel responses for COVID-19 impact mitigation in this community. Many of the emergency measures put in place by homeless service providers in Tippecanoe County, Indiana created opportunities for innovative solutions to longstanding challenges faced by homeless populations that are informing better service delivery moving forward, even beyond the COVID-19 pandemic.

**Conclusions:**

Community-based organizations, including homeless shelters, are uniquely qualified to inform pandemic response and disaster risk mitigation in order to respond appropriately to the specific needs of people experiencing homelessness. The lessons learned and shared by homeless service providers on the frontline during the COVID-19 pandemic have important implications to improve future disaster response for homeless and other vulnerable populations.

## Background

The COVID-19 pandemic has exposed and amplified the rampant health disparities and weaknesses of our public health system that inequitably impact marginalized and underserved populations in the United States (US), including people experiencing homelessness (PEH). Herein, we refer to homelessness not as a defining trait of an individual, but instead as a state that is experienced, one that is transitory and amenable to intervention. PEH face disproportionate rates of underlying health conditions and substance use disorders, stigma, and marginalization that often disenfranchise them from health and social services, and social living conditions that lead to a heightened risk of infection and adverse outcomes of COVID-19 [1–3]. Pandemic-related lockdown measures caused a sudden disruption in access to public spaces, restrooms, and other resources that PEH typically rely on to meet basic needs. Additionally, because of the economic consequences of the pandemic, growing rates of domestic violence [4], and the release of prisoners without social support or housing options [5], many communities throughout the US experienced increases in homelessness and demand for shelter beds. Homeless shelters throughout the country were reportedly overburdened and under-resourced to respond to this crisis, with drastic shortages of supplies and volunteers [6–8]. Furthermore, standard COVID-19 prevention guidelines, such as practicing social distancing, maintaining regular personal hygiene, and mask wearing can be difficult in congregate settings, placing homeless shelter guests, staff, and everyone they interact with at increased risk of infection.

Recognizing that PEH are particularly susceptible to COVID-19 infection and pose increased risk for community transmission, effective pandemic response efforts must prioritize these marginalized groups. Previous studies following natural disasters have found that PEH were often overlooked in disaster planning and response [9, 10]. Federal policy and funding were directed almost entirely towards homelessness prevention efforts and assistance for newly displaced individuals and families, leaving critical gaps in resources, communication and outreach programs for those who were already homeless prior to the disaster [9, 10]. While numerous resources rapidly become available for disaster response, homeless service providers generally lack a formal role in disaster planning and often lack established mechanisms to access relief resources or to assist PEH in doing so. Similarly during the COVID-19 pandemic, homeless service providers have been minimally involved in federal disaster planning, and coordination and reimbursement processes between government entities on disaster response is lacking [11, 12]. Four months after Congress passed the Coronavirus Aid, Relief, and Economic Security (CARES) Act, less than 30% of the $4 billion allocated to support homeless populations had actually reached those in need [13]. Furthermore, despite CDC recommendations for frequent COVID screening in shelters, most continuums of care across the US reported little or no testing capacity [14], and insufficient data to know who was getting sick and where [15].

At any given time, there are nearly 600,000 people around the country experiencing homelessness who sleep in temporary shelters or on the street [16]. The US Interagency Council on Homelessness estimated approximately 15,000 COVID-19 cases and 250 deaths among PEH in 2020 [17], but these are rough and incomplete estimates, and the full impact is largely unknown due to a lack of a centralized effort to track COVID-19 infections and deaths among the nation’s homeless population [15]. In Indiana, a point-in-time count in 2019 estimated nearly 5500 individuals experiencing homelessness in the state, although that number is reasonably underestimated because of the difficulties in the counting process [16]. To date, no statewide effort has been undertaken to track COVID-19 cases or deaths in Indiana’s homeless population specifically.

In Tippecanoe County, Indiana, a recent Community Health Needs Assessment Report cited homelessness and housing instability as community issues of highest concerns, with estimates of up to 900 homeless individuals in the county over a year, including an average of 180 homeless children reported by the Tippecanoe and Lafayette School Corporations [18]. In response, a growing number of community-based organizations and local programs have aimed at addressing these concerns, including a non-profit homeless engagement organization that serves as an initial point of entry for PEH in Tippecanoe County, which provides shelter, housing services and case management, three meals per day, and access to showers, phones, and toiletries to PEH, which they refer to as their “guests”. In partnership with this organization, an ongoing community-based participatory research (CBPR) [19] study is examining the challenges and impacts of the COVID-19 pandemic on PEH and homeless service providers in Tippecanoe County, Indiana. Guided by the socioecological model (SEM) [20], the findings presented herein document the challenges faced by PEH from the perspective of homeless service providers and the ways in which these providers prepared for, experienced, and responded to the pandemic for this vulnerable population.

## Methods

In congruence with the essence of CBPR, our community partner, a non-profit homeless engagement organization that serves as an initial point of entry for PEH in Tippecanoe County, contributed to all aspects of the study from formulation of research questions to identification of potential participants to the analysis and dissemination of findings. Our community partners were assumed most capable of providing the best accounting of influential homelessness service providers. As such, from July 2020 through January 2021 participants were recruited using quasi-snowball sampling [21], which involved initial contact with an existing community partner organization who provided a partial and initial list of relevant and established community-based organizations (CBOs) engaged with people experiencing homelessness in Tippecanoe county. As interviews were conducted, interviewees recommended additional local organizations and individuals of various levels of authority, whom were contacted and interviewed by the research team.

Academic and grey literature was reviewed to gain insights into the COVID-19 response among communities working with PEH and to identify knowledge gaps that could be addressed by the local CBO interviews. Informed by the SEM and the literature review, an initial interview guide was developed to understand multilevel challenges and responses to supporting PEH during the COVID-19 pandemic, from the perspective of local homeless service providers. Our community partner organization reviewed the interview guide and suggested additional questions and probes. The final interview guide included questions such as: “In your view, how did the day-to-day lives of PEH change as a result of the pandemic?”; “Did COVID-19 change the services or resources your organization typically provides?”; “Did your daily interactions and communication with PEH change as a result of the COVID-19 pandemic? If so, how?”; “Do you feel your organization was prepared to handle the pandemic?”; “Did COVID-related policy changes affect your organization? If so, how?”

In total, 18 semi-structured interviews were conducted with representatives of 15 organizations, including local government officials and a diverse array of CBOs within Tippecanoe County, Indiana. This included organizations that participated in the extended housing of the chronically homeless, emergency shelters for acute care, specialized homelessness services to those suffering from domestic violence, rapid re-housing and housing support programs, rental and foreclosure assistance programs, food banks, soup kitchens, mental healthcare, and other social service providers.

**Fig. 1:**
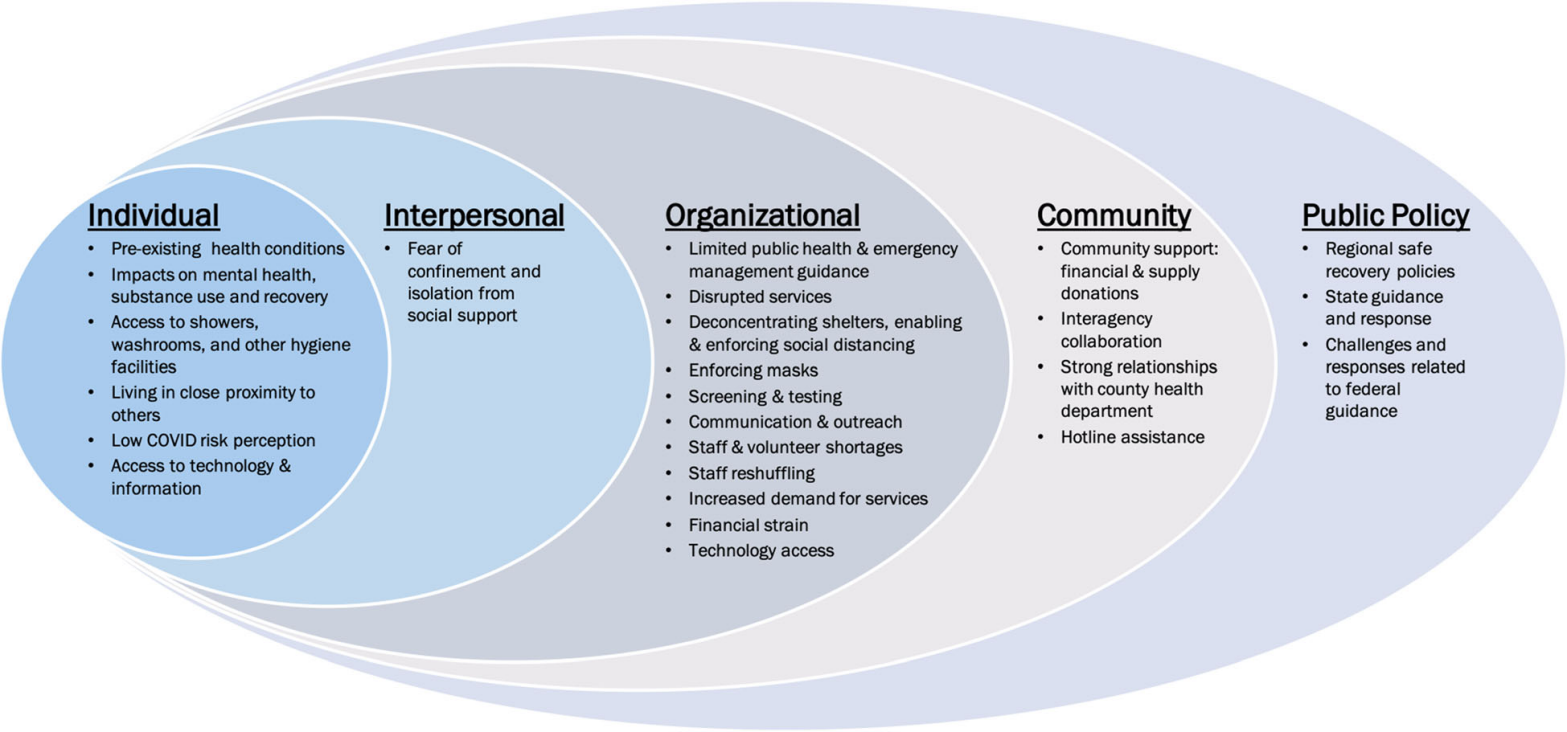
Socio-ecological model framework for multilevel challenges and responses to supporting PEH during the COVID-19 pandemic

Interviews were performed virtually by trained research assistants who were involved in formulating the research questions and in the grounded theory and associated constant comparative analysis; moreover, each interviewer had no prior association with any interviewed subject that could have biased the line of questioning or content of any given interview. Interviews were recorded and transcribed using Otter.ai, a digital scribing platform. Transcribed interviews were reviewed and edited for accuracy. Each interview was coded by multiple coders and subsequently discussed as a group to ensure intercoder consistency [22]. Utilizing a combinatorial approach of deductive and inductive coding, data was thematically analyzed [ 23] using NVivo, a qualitative coding software [24]. Guided by the SEM, themes were organized across individual-, interpersonal-, organizational-, community-, and public policy-levels. (Fig. 1).

As part of the CBPR approach, preliminary findings that highlighted the strengths, opportunities, and challenges of the community-based responses to COVID-19 were shared with the primary community-based partner, as well as a local network of homeless service provider organizations to incorporate their feedback and insights and ensure community involvement in all aspects of the research and analysis. This study was approved by Purdue University’s Institutional Review Board (protocol IRB-2020-1005).

## Results

### Individual-level challenges

Organizational staff provided powerful examples of the concerns they had for homeless clients. They discussed their clients’ poor health and the pre-existing conditions that magnified their risk for COVID-19: “*Especially with this population, like not only do they not have homes, they all have horrible health … while [other organizations] have a kind of targeted criteria for those that are like at high risk for COVID; well that’s almost everybody in my day room.*” Staff also worried about the impact of prolonged shutdown periods and disruption to routines on clients’ mental health, “*in general, the population was a lot more stressed... services weren’t necessarily available to them. The isolation is a huge problem [and] all their regular places to hang out were kind of shut down and dispersed. The isolation really had a negative impact on their mental health.”* Staff also expressed concerns related to reductions in access to substance addiction treatment services and counseling and how these circumstances negatively impact their clients’ mental health, substance use, and even relapse. *“We work closely with an organization that provides mental health care. But during this whole pandemic … they weren’t coming to the building … mental health care is a critical piece of programming, quite frankly, and to have that go away, coupled with all the changes and challenges, it was awful … we saw a lot of behaviors that were a result of [clients] not being able to access that care … the substance use is out of control …*”.

In regards to COVID-safety measures, staff members described how difficult it was for clients’ to engage in basic hygiene practices like handwashing due to lack of access to showers and washrooms, *“those living unsheltered really needed access to like hygiene stations because a lot of … businesses and places that [homeless] people normally go … were not available during the pandemic.”* Closures also resulted in the loss of other critical resources such as clothing, meals, and food donations and as a result, “*… they just ran out of places to go. There wasn’t many places for people to be and feel safe, be and feel comfortable …”* Living in congregate shelter settings impeded adoption of COVID-safety practices, *“… they’re all together, they all share cigarettes, they all share food, they’re all touching each other.”* Also, general disbelief and low COVID risk perception heightened individual’s risk, *“… they just don’t pay any attention to it. They don’t think it’s real. We even had the health department come down here. You know and talk to them, but I don’t know, they just don’t think that it’s going to affect them.”* Staff explained that their clients’ fears of being confined, alone, or separated from their social group led many to reject quarantine practices, “*the period of time where we were locked down and they really couldn’t go anywhere, you know, they weren’t supposed to be going to the convenience store or anything like that, they did not like that. They don’t like being confined* …” This was further illustrated by another staff member who shared, “[some] … *could have used the shelter [but] chose not to because they didn’t like the idea of quarantine to begin with and then to do it in a hotel, they didn’t really like that idea either because you’d have to be separated from friends and family for two weeks. And so they just figured out a way to quarantine some other way.*”

Even when restricted or adapted services became available, clients continued to face challenges. For instance, a major early development was the provision of virtual services; unfortunately, barriers to accessing these modified services persisted for clients’ with poor telephone or computer access, “o*nce COVID hit, it was very interesting to see our partners pivot, because they all shifted to work from home and they would do telehealth … Well our people don’t have telephones, they’re not calling and scheduling appointments. It was really awful.”* Moreover, when efforts were made to provide clients phones, these same clients lacked access to settings that would allow them to charge or store the devices, “*we connected people to those resources so we help people you know get an email address, if they have the opportunity to have a computer, we help people get the free phones, so that people do have access to a phone to call people. But our people don’t have a place to charge them, and they don’t have a computer … the pandemic added a whole new set of challenges.”* Staff also recognized that clients’ limited access to information created challenges, “*they’re not connected to the news and social media, the way we are. They don’t have Facebook on their phone, they don’t have Twitter, they’re not looking at all of that.”* They went on to discuss the role organizations failed to play in providing clients information, “*we don’t have TVs or anything here so if they don’t have phones where they can get online and see what’s going on, they really don’t know what’s going on in the outside world and they really don’t care.”*

### Organizational-level challenges and responses

Several organizations had policies and procedures in place for natural disasters like tornadoes and earthquakes, armed intruders, bed bugs and lice, and flu outbreaks. However, most felt there was no precedent for this kind of pandemic response in homeless populations. “*With COVID there was no rulebook whatsoever … We had no idea what we were getting into, no one did*.” Nevertheless, most participants felt their organizations were able to quickly respond, leaning on local partners and state guidance to adapt procedures and implement additional precautions in order to stay open and continue to serve the homeless community. “*I feel like no one was ready for this, right? But we were not completely flat footed. There was a pretty nimble response to the reality, in my opinion, and we didn’t miss a beat, never closed the shelter for a single moment. Everything continued*.”

Staff described initial confusion and uncertainty around how best to implement COVID-19 safety measures in crowded homeless shelters. Some organizations implemented an initial shutdown where they did not let anyone new into the shelter for a period of time. Other organizations suspended services, which created challenges to reaching and staying connected to clients. *“Well, for quite a long period of time, we were not allowed to [bring] people in our office … So we could get on the phone with them and they would be out at the front desk, but then there’s the whole privacy thing … If they’re not comfortable saying over the phone what they need to talk about, then, it was really hard to do anything. So, it did affect their services for a period of time.”* Some organizational staff expressed concerns that these service changes would lead to negative outcomes such as health complications for clients who require on-going care. “*Basically, if we can’t see them, and they don’t have a phone, and we don’t know how to get a hold of them by phone. We couldn’t do med management with them. We couldn’t do therapy with them and we couldn’t do much for case management, so we didn’t. We didn’t do anything for some clients. I mean it put everything on halt for some people that had no other resources or couldn’t get here on their own.”*

New safety procedures and reminders were implemented to promote physical distancing in the shelter and during meals. *“One thing that we have continually had to emphasize is the number of people in our community space … social distancing in a really large day room, that’s a challenge … we had to frequently do reminders … constantly having to tell people to spread out, you need to be six feet apart.. we do our best to keep socially distant during meal times … we’ve staggered our seating and our tables and things like that. And we’ve also modified the way that we serve dinner, most things are now self-serve so there’s limited contact between volunteers and staff and the guests.”* Moreover, organizational staff began to offer COVID-related communications including information related to new procedures and expectations for homeless clients through face-to-face group announcements in the shelter. *“We try and make announcements out in our day room where everybody is … had a little town hall meeting with folks … you talk this through and try to explain what the situation is and why we have these expectations.”* These modifications also necessitated that COVID practices be enforced which the staff found challenging.*“We have people trying to sneak past the front desk and get their temperature taken and stuff like that.”*

Staff discussed difficulties particularly around enforcing physical distancing and masks among homeless clients. *“Trying to keep people apart here in our facility is pretty horrible, you know, keeping the mask on and keeping people six feet apart, that’s been a huge problem for us.”* As a result, some organizations were forced to reduce or discontinue essential services such as meal services. “*It wasn’t [possible to have evening meal]. We weren’t able to have everybody six feet apart the whole time and you’re taking off your mask to eat and there’s 20 people in a room we’re not supposed to have a gathering at all. So, some of those things we just weren’t able to do.”*

In response, some organizations implemented very strict mask policies, while others concluded that it would be too difficult to enforce and thus focused efforts primarily around requiring staff to wear masks. *“We can’t discipline everyone … that’s not about who we are and what we do. So, our approach in general, pandemic or no, is to give people the opportunity to make a better choice tomorrow. So that’s how we roll. We take as many steps as we can … staff wearing masks and six feet apart and all of those kinds of things but if it comes down to people being here and not wearing a mask or people being on the street and not wearing a mask, we want them to be here where they’re receiving services.”*

Limited options for screening and testing were discussed as key challenges to identifying cases and preventing further transmission. *“Some of the challenges that we have encountered are getting our people tested. Right now, the only avenue that we have is to call an ambulance and have them tested through the emergency room. And then when they return, we have to isolate them until their test comes back … But a lot of our people have been sitting in the same room together … they’ve already been exposed to each other. Getting test results back has been a huge issue.”* In response, some shelters implemented temperature checks and symptom screening at the door. They also relied on the health department and local hospitals to provide testing for homeless clients. *“When people come in, their temperatures are taken … and if anyone has a fever that’s like over 100, we contact the health department to see if they need to go be tested for COVID [at a hospital] … And if they are positive, they will be taken to a hotel by the health department for however long their isolation period is, and then they come back.”*

The crisis led to operational challenges due to declines in staff levels and available volunteers. “*Some organizations rely really heavily on, mostly volunteers, and a lot of volunteers tend to be maybe like older folks or retired folks who are then in high-risk categories. And so they lost pretty much their entire volunteer base.”* Likewise, challenges emerged related to lack of organizational staff policies that “*… certainly affected our staff, because I didn’t we, we put into place, like a COVID sick time policy, so if you or a family member or your children are in school or if you have some compromised immune system, I expected you to work from home. You know what I mean like that’s the deal. Now did every one of my employees work eight hours a day from home? No, no. So that certainly was unfortunate.”* In response, organizations implemented staffing reorganization plans to enhance staff safety and to offset the sudden lack of volunteers. *“We also then split our staff team up into, initially it was three different groups, so we would do one week [at home] and three in the shelter. And that was including staff that don’t usually work in our shelter … because we couldn’t have volunteers anymore.”*

Besides the strained staffing levels, many organizations faced serious financial challenges. At the start of the pandemic organizations lost donors, “*anytime there’s some sort of massive upheaval in the social fabric of the U.S., it’s always the nonprofits … that have the most sustained reaction to that because more and more people will continue to come into services. [However], fewer and fewer people will be in a position to donate.”* This was further illustrated, “*our fundraiser in August is canceled. Our fundraiser in April didn’t do really well”.* Organizations also experienced unexpected expenses associated with having to purchase COVID protection equipment and supplies for staff and clients. “*We had to get all the PPE, I mean I just bought 1000 masks yesterday, you know none of these things were ever in our budget before to buy plexiglass for 1000 bucks and I’ve got meals now being delivered from a hospital. We’re having meals catered from them, that’s another $4,000 expense a month.* These financial strains were made worse as organizations attempted to adapt their services for a virtual platform, *“to use a platform like [zoom] we [need] to get new computers new webcams new platforms.”*

Amid this backdrop of operational challenges, homeless shelters also experienced pressures related to increasing demands and needs for beds. *“You know earlier in COVID before that unemployment benefit kicked in, we were seeing more households than before. And in early COVID they were higher than they are now because people couldn’t go out and buy their own groceries. So, we are concerned that not receiving as much in income we’ll see more demand.”* An organizational strategy to address this increased demand was to pay for hotel rooms for high-risk clients and those soon to be housed. *“Our most at-risk population plus those that were on a path to housing, we just went ahead and paid for them to get out of here to just deconcentrate the day center … we put them into a hotel until they were able to leave the hotel and go into housing.”*

### Community-level challenges and responses

Most participants expressed that the community response to organizational- and individual-level challenges was overwhelmingly positive. Many discussed the tremendous support received from community members, faith-based organizations, local businesses, and donors. *“We had a tremendous outpouring of support from local businesses and the United Way, and individual donors.” “We have a very big community of people that anytime we need anything, we just put the word out on Facebook, and we have multiple people that will bring things in, donate money, do whatever we need...”*

Participants also described the support and guidance they received from established interagency relationships and collaboration. *“In our community, we work well together, that doesn’t happen everywhere … we’re pretty lucky … the relationships that helped us early on were with each other …” “The Homeless Prevention Intervention Network, which is all of the agencies that have some touch with this population … we’re co-providing services, we share the same clients … there’s a monthly meeting and there’s pretty strong communication. We had been meeting monthly for years. So when all this was kind of coming down the pike, you know, we were already meeting, we were already talking.”* One such coordinated effort among the agencies was the creation of a ‘Housing Instability Hotline’ to connect people economically impacted by the pandemic to rental assistance and other resources. *“The hotline did not have any unique resources to … we have like 15 navigators that were trained on how to answer those calls, all of them are volunteers, all of them came from other agencies so all the agencies came together to create this pool of people to answer the calls … [this was] just a real important strategy I think at the beginning.”* The existing interagency collaborations also enabled rapid coordination of efforts during outbreak-related shelter shutdowns. *“When the shutdown happens, it prevents any new people from coming in. The community put together what’s called an Annex, a safe space [one organization’s empty gym], which provides shelter for people to come in at different periods throughout the day … they come in and they get snacks, they can take a shower, they’ve got cots that they can lay and rest on … The Annex is a cooperation between a lot of different agencies in Lafayette.”* Participants also described strong partnerships with the local health department which provided a constant source of guidance. *“We have a very good partnership with the Tippecanoe County Health Department and so they were here, often, and were informative …*. *we have been in constant contact.”*

### Policy-level challenges and responses

Participants expressed frustration over a lack of federal guidance, especially in the early days of the pandemic. *“One of the frustrating things about it, in the beginning there was no clear guideline. So constantly watching our local government web pages, trying to reach out to those resources … using the information [from] CDC, different webinars and seminars, a lot of my time was devoted to researching and finding things on my own and kind of being in the know and joining group chats and countless zoom meetings to find out the latest information, because in the beginning, there was no clear protocol, and even now, things are changing daily.”* Additionally, participants felt that several aspects of federal and state guidance, such as ‘stay at home orders’, was not appropriately aligned with the context and realities faced by homeless populations. “*It’s hard to enforce that with people living outside when they didn’t even have access to places to wash their hands, or use the restroom … those are really hard things, I think, too, to socialize and adhere to when you’re not living housed and you don’t have access to the same resources that people in housing do*.” Moreover, many felt that most of the community-based organizations’ funding for pandemic response was from local sources and that there was an overall lack of federal response funding for the local homeless population. *“It’s all local, so these are local donors like private individuals and companies. We have not received any federal money yet …”.*

Where federal response and guidance were lacking, many participants felt that the state response stepped in to fill those gaps. *“We had a lot of support from our local health department, but also at a state level, like in the early days of COVID advice from some of our state base and state housing organizations.” “The governor putting a hold on evictions and foreclosures was a good thing … we needed to take a minute to figure out, you know, what’s going on here. And I’ve been glad about that, that that happened.”* The state of Indiana also coordinated regional safe recovery sites, where homeless individuals awaiting COVID-19 test results or needing to quarantine could stay, and encouraged inter-region communication, which many participants appreciated. *“Inter-region communication has increased dramatically. We rarely talk to other regions … But we have weekly calls on the safe recovery site which includes all the regions … about what they’re doing in terms of homelessness, which has been really helpful … just hearing what those folks are doing and describing what they’re going through has been really helpful.”*

### Lessons learned and silver linings

In the midst of overwhelming challenges of the COVID-19 public health crisis, participants also shared important lessons learned in the process of quickly adapting their service delivery. In fact, many of the emergency measures put in place by homeless service providers created opportunities for innovative solutions to longstanding challenges faced by homeless populations that can inform better service delivery moving forward. Table 1 outlines some of the key lessons that community-based homeless service providers felt were important to implement for improved pandemic and post-pandemic response for people experiencing homelessness.

**Table 1:** Key Lessons for Pandemic Response in Homeless Populations

Key Lessons	Participant quote(s)
1. Implement/ strengthen interagency community partnerships.	*“I think another thing that we’ve put in place during this that we want to continue is just the way we’re coming together as a community to problem solve and work together. So, these weekly calls emerged out of a crisis response, but it’s really kind of built a new sense of community and trust and transparency among lots of different partners that I think we will continue well beyond this.”*
2. Provide mental health and substance use services in homeless settings.	*“We definitely saw a shift in the need during COVID-19 of expanding access to behavioral health services, specifically around connections to mental health services and substance abuse treatment.”*
3. Maintain and expand telehealth access.	*“A lot of health providers are saying like hey telehealth is actually a pretty good way to connect with people. And so we’re trying to figure out how to continue building the infrastructure to support telehealth for people experiencing homelessness or newly housed to be able to continue to access their healthcare in that kind of way, and not have to physically go to a location to see a provider.”*
4. Track and share data to better inform practices.	*“We’ve been forced to do some more data sharing and data tracking to really understand the impact of COVID-19 particularly on those experiencing homelessness … to do data tracking in a way to help inform our community and to make informed practice is something I think that we’re really learning through this process of the importance of being able to understand in as real time as possible, how things are affecting those experiencing homelessness. So that’s been, I think, an important practice that we will absolutely need to continue to improve on.”*
5. Expand targeted outreach strategies.	*“The way in which, for example, we’re doing street outreach and things like that … outreach teams have been amazing … coordinating crisis response to making sure those living unsheltered really have access to not only their basic needs but then access to getting connected to resources and hopefully housed, and making sure like the whole city is covered in terms of where there might be pockets of unhoused people. And so I think that’s a practice that we’re definitely looking at continuing and scaling doing outreach in a much more targeted and dedicated way.”*
6. Increase shelter diversion resources.	*“For the shelter, I would really love to keep a reduced capacity, because I think it’s more trauma informed … I think it’s undignified to have to share space with someone else, particularly if you’re going through trauma. So I’d love to keep that but the only way to keep that would be to keep up the increased amount of resources we have for shelter diversion like hotel rooms and deposit first month’s rent and rental assistance.”*
7. Reprioritize ending, not just preventing, homelessness.	*“During COVID-19, I think we’ve also just seen the importance of, and demand for permanent housing. It’s really hard to keep people safe and healthy in congregate shelters or living in unsheltered locations.”* *“So, there’s a difference between preventing eviction and preventing homelessness … what about all these people that don’t have housing [now]. I just wish we could reprioritize what matters because I’ve seen what happens when people experience homelessness, and how their health, both physical and mental deteriorate after a while living in a homeless shelter or on the street, it is absolutely horrendous to see what happens to people.”*
8. Prepare for long-term impact mitigation.	*“I think that people understand that COVID might end tomorrow, but our work is not going to end tomorrow, people are going to struggle with this for a long time, because you don’t lose that much income and come right back from it... So it’s going to be long term. We’re going to have to be willing, as a nation, to help people for a long time.”*
9. Leverage the COVID-19 crisis to increase visibility of homelessness.	*“If there was a silver lining in all of this, I would say that what COVID has done for those who are experiencing homelessness … like people now kind of care, right, because we’re in the middle of a pandemic and … we’ve got these people that can’t shelter in place and they can’t protect themselves and they’re perhaps, they’re out spreading this virus to everyone as a public health crisis, or they’re all going to die. So, what, wait a second, is that okay? no it’s not okay. And so, it’s been interesting to see from a local, state, and national level the type of conversations that are being had that homelessness is not okay. Like what we’ve allowed as a country for these people that have happened to them is not okay.”*

## Discussion

This study examined the impacts of the COVID-19 pandemic on people experiencing homelessness in Tippecanoe County, Indiana and the experiences, challenges, and responses of homeless service providers. The socio-ecological model guided the analysis of multilevel challenges and responses for COVID-19 risk and impact mitigation for this homeless population.

Homeless service providers identified challenges at the individual level including the disproportionate risks and vulnerability of this population due to pre-existing physical and mental health issues, substance use prevalence, limited access to basic needs, healthcare services, and education. While many of these are social determinants of health indicative of structural issues and inequities, they were presented as individual-level challenges by our participants because they are experienced most directly by PEH. These identified challenges echo existing literature on homelessness and health [1, 2, 25]. The disruption of in-person services for mental healthcare and addiction recovery amplified many of these issues for this population. Providers shared that PEH have minimal access to technology or reliable communication channels that led to a lack of information and understanding of the pandemic. As a result, disbelief and low risk perception among homeless individuals led to an overall reluctance to comply with COVID-19 safety measures, as reported by shelter and other organizational staff. Moreover, provider perspectives offered insights into the need to understand how strategies and policies might further marginalize or traumatize this population. For instance, quarantining practices were rejected by clients because of confinement fears and concerns related to being separated from their social group not solely due to lack of compliance.

In alignment with numerous reports around the US [16, 26], homeless shelters and other organizations reported increased demand for homeless services due to the pandemic, and numerous operational challenges including loss of volunteers and staffing issues, additional unbudgeted expenses for PPE, and difficulty deconcentrating spaces or enforcing masks and social distancing. Community-based homeless service organizations described frustrations around lack of federal guidance and challenges navigating emergency relief resources and funding. Guidelines around reduced shelter capacity and frequent testing were largely infeasible for many organizations that lacked additional resources or mechanisms for shelter diversion or rapid testing. Additionally, federal guidance lacked adequate regard for this population’s vulnerability, context, and ability to adhere to recommended COVID safety measures.

These challenges were not unique to this community or to this disaster, and our findings provide further evidence of an overall neglect of homeless populations in disaster preparedness and response [27]. Whereas disaster response has often focused on homelessness prevention or on providing housing and assistance for those displaced during the event, it has often overlooked individuals and families who were already homeless prior to the disaster [27]. The experiences shared by Tippecanoe County homeless service providers further support these reports.

Despite numerous reported challenges, participants also shared the myriad ways this community came together to respond to this unprecedented public health crisis for a vulnerable homeless population. Organizations leaned heavily on each other to share experiences and best practices. Interagency collaborations enabled rapid implementation of coordinated response efforts for community assistance, resource navigation, and continued provision of basic services during periods of shelter shutdowns. Participants stressed the importance of these strong multisectoral partnerships as being key to effective pandemic response for this vulnerable population because the challenges spanned issues related to housing, health, law enforcement, among other sectors.

Unlike major homeless shelter outbreaks reported elsewhere [2, 3, 7, 28], as of February 2021, the total number of confirmed positive COVID-19 cases among people experiencing homelessness in Tippecanoe County was estimated to be less than 30, and no COVID-related deaths were reported among homeless individuals in the county. Overall, homeless service providers were able to meet the basic needs of homeless individuals while avoiding major outbreaks or total shutdowns. The lessons they learned in the process are invaluable to informing future pandemic response for homeless populations. Furthermore, many ways in which they adapted their practices could improve service delivery for homeless populations long after the COVID-19 pandemic.

While the unique perspectives of the service providers in this study offer key lessons for pandemic response in homeless populations, a limitation of this present study is that it does not include the perspective of people experiencing homelessness. Future work will focus on homeless community member narratives around their individual experiences during COVID-19, including their awareness and perceptions of the disease, risk factors, and prevention measures, their perspective on local response efforts, what they believe their own needs are, and how they believe those needs should be met.

## Conclusion

The particular vulnerability of PEH and consequently the increased risk for PEH to contribute to community transmission of COVID-19 should have prioritized these populations in pandemic response and relief efforts. This has not been the case in most communities throughout the U.S., and one reason for the exclusion of these groups in general health promotion programs has been a common inability to engage these ‘hard-to-reach’ populations [9, 29]. Robust evidence regarding the ability to engage with homeless communities is essential to inform policy and practice to improve public health outcomes and to inform targeted pandemic response efforts in the future. To effectively reach these populations, initiatives should be based on the voices of the affected and the guidance and input from community-based organizations and leaders who have knowledge of the needs and available resources within vulnerable communities.

Community-based organizations, including homeless shelters, are uniquely qualified to inform, and should be included in planning efforts for, pandemic response. Homelessness is a result of varying circumstances for a wide range of people, thus there is no one-size-fits all approach and pandemic response and impact mitigation strategies must be tailored to specific local contexts [30]. Disaster response in general must be more inclusive and recognize the unique circumstances of PEH within the context of public health disasters in order to respond appropriately to their needs. The lessons learned and shared by CBOs on the frontline during the COVID-19 pandemic have important implications to improve future disaster response for homeless and other vulnerable populations.

## Data Availability

The datasets used and/or analyzed during the current study are available from the corresponding author on reasonable request.

## Abbreviations

CARES: Coronavirus Aid, Relief, and Economic Security
CBO: Community-based organizations
CBPR: Community-based participatory research
PEH: People experiencing homelessness
SEM: Socioecological model
